# Co-targeting EGFR and mTOR with gefitinib and everolimus in triple-negative breast cancer cells

**DOI:** 10.1038/s41598-020-63310-2

**Published:** 2020-04-14

**Authors:** Abderrahim El Guerrab, Mahchid Bamdad, Yves-Jean Bignon, Frédérique Penault-Llorca, Corinne Aubel

**Affiliations:** 1 Université Clermont Auvergne, Faculté de médecine, INSERM, U1240, Imagerie Moléculaire et Stratégies Théranostiques, 28 place Henri Dunant, BP38-, 63001 Clermont, Ferrand France; 2 Université Clermont Auvergne, Institut Universitaire de Technologie de Clermont-Ferrand, Département Génie Biologique, Ensemble Universitaire des Cézeaux, CS 30086-, 63172 Aubière Cedex, France; 3 Centre Jean Perrin, INSERM, U1240, Imagerie Moléculaire et Stratégies Théranostiques, 58 rue Montalembert, BP392, 63011 Clermont, Ferrand Cedex France

**Keywords:** Breast cancer, Breast cancer

## Abstract

Triple-negative breast cancers (TNBC) are unlikely to respond to hormonal therapies and anti-HER2-targeted therapies. TNBCs overexpress EGFR and exhibit constitutive activation of the PI3K/AKT/mTOR signalling pathway. We hypothesized that simultaneously blocking EGFR and mTOR could be a potential therapeutic strategy for the treatment of TNBC. We examined the antitumour activity of the mTOR inhibitor everolimus combined with the EGFR tyrosine kinase inhibitor gefitinib in TNBC cell with or without activating mutations in the PI3K/AKT/mTOR signalling pathway. We demonstrated that everolimus and gefitinib induced synergistic growth inhibition in the *PI3K* and *PTEN*-mutant CAL-51 cell line but not in the PTEN-null HCC-1937 cell line. The antiproliferative effect was associated with synergistic inhibition of mTOR and P70S6K phosphorylation, as well as a significant reduction in 4E-BP1 activation in the CAL-51 cell line. We also showed that combination therapy significantly inhibited cell cycle progression and increased apoptosis in this cell line. Gene and protein expression analysis revealed significant downregulation of cell cycle regulators after exposure to combined treatment. Collectively, these results suggested that dual inhibition of mTOR and EGFR may be an effective treatment for TNBC with activating mutations of *PI3K*.

## Introduction

In recent decades, clinical, immunohistochemical, and molecular studies have been used to divide breast cancer into several subtypes^1^. Among these multiple subtypes, triple-negative breast cancer (TNBC) is defined by reduced expression of hormone receptors and human epidermal growth factor type 2 receptor (HER2). This type of breast cancer is unlikely to respond to hormonal therapies and anti-HER2-targeted therapies. Chemotherapy and radiation therapy are currently the only ways to treat TNBC patients, but these treatments provide a partial response with early relapse and worse prognosis^2^. Clinical studies have shown that TNBC is more likely to recur in the first five years after treatment and develop multidrug resistance to chemotherapy^3,4^. No second-line targeted therapy is currently approved for this type of cancer.

The majority of TNBC cases are characterized by the overexpression of a receptor tyrosine kinase: epidermal growth factor receptor (EGFR). This feature has emerged as a potential therapeutic target^5^. It has been reported that the frequency of EGFR overexpression in TNBC is as high as 76%, suggesting that the large majority of TNBCs are likely to benefit from anti-EGFR targeted therapies^6,7^. Among the different EGFR inhibitors currently approved in oncology, gefitinib is a selective EGFR tyrosine kinase inhibitor (EGFR-TKI) indicated for the treatment of patients with advanced or metastatic non-small-cell lung cancer (NSCLC) harbouring activating mutations of *EGFR*^8^. Despite the absence of *EGFR* mutations in TNBC, gefitinib has been evaluated in TNBC patients. Clinical studies have reported that gefitinib enhanced the growth inhibitory effect of chemotherapies, but the use of gefitinib alone failed to demonstrate significant efficacy^9,10^. These disappointing results could be related to the molecular heterogeneity of TNBC, characterized by diverse genetic alterations in EGFR signalling pathways.

Triple-negative tumours with overexpression of EGFR exhibit constitutive activation of EGFR-dependent signalling pathways, especially the PI3K/AKT/mTOR pathway. Activation of this pathway is involved in tumorigenesis, contributing to apoptosis inhibition, cell cycle progression, drug resistance, cell motility and metastasis^11,12^. Several molecular alterations affecting the key components of the PI3K/AKT/mTOR signalling pathway are frequently encountered in TNBC. Among these genetic aberrations, the loss of *PTEN* expression and the presence of activating mutations in the gene encoding the catalytic subunit alpha of PI3K (*PI3KCA*) are responsible for the constitutive activation of downstream effectors such as mammalian target of rapamycin (mTOR)^13,14^. mTOR is a serine/threonine kinase that is directly phosphorylated and activated by AKT. Activation of mTOR controls the translation of mRNAs through the activation of eukaryotic translational initiation factor 4E-binding protein (4E-BP1) and ribosomal protein S6 kinase (P70S6K)^15^.

Preclinical studies have suggested that targeting mTOR could improve the efficacy of EGFR inhibitors in various human cancers, including TNBC. Everolimus is a rapamycin analogue that is currently used for the treatment of several solid malignancies including renal cell carcinoma, neuroendocrine carcinoma and HER2-negative breast cancer^16–19^. An *in vitro* study demonstrated that everolimus and gefitinib induced synergistic growth inhibition of EGFR wild-type NSCLC cell lines^20^. Another study demonstrated that everolimus restores gefitinib sensitivity in resistant NSCLC cell lines. Everolimus plus gefitinib induced a significant decrease in the activation of EGFR downstream signalling pathways and resulted in a synergistic growth-inhibitory effect in NSCLC cells^21^. Reports from other authors showed that combination of EGFR and mTOR inhibitors synergistically inhibits the cell cycle progression and the growth of several colorectal carcinoma cell lines^22^. Liu et *al*. showed that co-inhibition of mTOR and EGFR (using rapamycin and the EGFR-TKI lapatinib, respectively) resulted in synergistic effects in TNBC cell lines and suppressed triple-negative tumour growth *in vivo*^23^.

Collectively, these studies provide evidence that the approach of co-targeting mTOR and EGFR could be a potential therapeutic strategy in TNBC. In the current study, we hypothesized that inhibition of mTOR may improve the efficacy of anti-EGFR targeted therapy in TNBC with molecular alterations of PI3K/AKT/mTOR pathway. We investigated the combination effect of everolimus and gefitinib in several TNBC cell lines that do or do not harbour the main activating mutations in the PI3K/AKT/mTOR signalling pathway. We used cell lines overexpressing EGFR and carrying *PI3K* and/or *PTEN* mutations, which are the most frequently encountered mutations in TNBC. We examined the effects of therapies in order to evaluate the therapeutic response according to these genetic alterations. We analysed the effect of gefitinib and everolimus on cell proliferation, cell cycle, apoptosis and expression of various genes involved in the process of tumorigenesis.

## Methods

### Cell lines, culture conditions and reagents

HCC-1937 (CRL-2336), SUM-1315 (SUM1315M02) and CAL-51 (ACC-302) cell lines were purchased from the American Type Culture Collection (ATCC, Manassas, VA, USA), Asterand (Detroit, MI, USA) and DSMZ (Braunschweig, Germany), respectively. All cell lines are triple-negative breast cancer cells and were conserved in the Biological Resource Center of Jean Perrin Comprehensive Cancer Center (No. BB-0033-00075, Clermont-Ferrand, France) (Table 1)^24,25^. Cells were cultured as described previously at 37 °C in a humidified atmosphere of 95% air and 5% CO2^26,27^. HCC-1937 cells were cultured in RPMI 1640 and CAL-51 in DMEM medium (Invitrogen Life Technologies, Carlsbad, CA, USA). The media were supplemented with 10% heat-inactivated foetal bovine serum (FBS), 2 mM L-glutamine and 20 mg/mL gentamicin. SUM-1315 cells were cultured in Ham’s F-12 medium supplemented with 5% FBS, 1% HEPES buffer, 10 ng/ml EGF and 5 μg/ml insulin (Invitrogen Life Technologies, Carlsbad, CA, USA). The EGFR tyrosine kinase inhibitor gefitinib and the mTOR inhibitor everolimus were purchased from LC Laboratories (Woburn, MA, USA). Drugs were dissolved in DMSO and stored at −20 °C. Dilutions were made immediately before use in growth medium, and cells were treated with various concentrations of drugs for 24 h, 48 h or 72 h. The final DMSO concentration (0.2%) remained constant in all analysed cell cultures, including untreated cells.

**Table 1:** Characteristics of triple-negative breast cancer cell lines used in this study.

Cell lines	TNBC Subtype	Basal subtype	Mutations
**CAL-51**	Mesenchymal-like	Unclassified	*PI3KCA, PTEN, MAP3K1*
**HCC-1937**	Basal-like 1	Basal-like A	*PTEN, BRCA1, TP53*
**SUM-1315**	Unclassified	Basal-like B	*BRCA1, TP53, CDKN2A*

*Data were obtained from references*^24,25^ *and* COSMIC database *(*www.sanger.ac.uk/genetics/CGP/cosmic/*)*.

### Cell viability assay

Cell viability was assessed using the XTT Cell Viability Assay Kit, according to the instructions of the manufacturer (Biotium Inc., Hayward, CA, USA). This method was described in our previous study^27^. Briefly, after determining optimal starting cell density for each cell line (densities between 5000 and 15000 cells per well), cells were seeded in sextuplicate in 96-well plates and incubated overnight. After attachment (24 h), cells were treated for 72 h with increasing concentrations of gefitinib (1, 5, 10, 20 and 50 μM) and everolimus (0.1, 1, 10, 100 and 1000 nM) as single agents and in combination. Fifty microliters of the activated XTT solution was added to the cultured cells in each well. Cells were incubated at 37 °C for 4 h, and the absorbance signal was measured with a spectrophotometer at a wavelength of 450–500 nm. All experiments were performed in triplicate. The relative cell viability was expressed as percentage of that of the untreated cells, and the IC50 values were determined by linear extrapolation. The combined effect of gefitinib and everolimus was determined using the Bliss independence model which allows the calculation of the expected effect of combination therapy (additive, synergistic or antagonistic effect)^28^. We described this model in our previous studies^26,27^.

### Western blotting

Immunoblotting was performed as described previously^26,27^. Briefly, cells were cultured in 100-mm dishes at a density of 5 × 10^5^ cells per dish and treated with 5 μM gefitinib and/or 100 nM everolimus for 24 h or 48 h. Twenty micrograms of each protein samples from cell lysates were separated via 7–12% SDS-PAGE (Bio-Rad, Hercules, CA, USA) and transferred to PVDF membranes (GE Healthcare, Westborough, MA, US). Blots were blocked and incubated overnight at 4 °C with the following primary antibodies: anti-phospho-ERK1/2 (Thr 202, Tyr 204), anti-ERK1/2, anti-phospho-AKT (Ser 473), anti-AKT, anti-phospho-mTOR (Ser 2448), anti-mTOR, anti-phospho-P70S6K (Thr 389), anti-P70S6K, anti-phospho-4E-BP1 (Thr 37, Thr 46), anti-4E-BP1, anti-EGFR, anti-IGFR, anti-CCNE1, anti-CCNB1 and anti-CDKN1C at final dilutions of 1:1000 (Cell Signalling Technology, Danvers, MA, USA) and anti-β-actin at final dilution of 1:5000 (Calbiochem, San Diego, CA, USA). Western blots signals were visualized using a chemiluminescence kit (Amersham Bioscience, Piscataway, NJ, USA) and quantified using ImageJ software. The intensity of individual bands was expressed relative to the control signal.

### Cell cycle analysis

Cell cycle analysis was carried out as described in our previous studies^26,27^. Briefly, cells were cultured in 6-well plates with 5 × 10^4^ cells per well and treated with 5 μM gefitinib and/or 100 nM everolimus for 48 h. Non-adherent cells were collected, and adherent cells were washed twice with cold PBS, harvested by trypsinization and centrifuged at 500 g for 10 min. Cell pellets were washed with PBS, and cell membranes were disrupted by repeated cycles of freezing and thawing in liquid nitrogen. Then, cells were resuspended in 200 μl of ribonuclease A solution (1 mg/ml) and stained with 200 μl of propidium iodide solution at a final concentration of 50 μg/ml (Sigma-Aldrich, St Louis, MO, USA). Fluorescence of cells was analysed by flow cytometry on a Cytomics FC 500 MPL Flow Cytometer (Beckman Coulter, Brea, CA, USA), and the percentage of cells at G0-G1, S and G2-M phases was determined using ModFit LT 2.0 software (Verity Software House, Topsham, ME, USA).

### Apoptosis assay

Apoptosis was analysed with FITC Annexin V Apoptosis Detection Kit I (BD Biosciences, San Diego, CA, USA), according to manufacturer’s protocol as described previously^26,27^. Cell preparation and treatments were performed as in the cell cycle experiments. After centrifugation, cell pellets were resuspended in 100 μl of binding buffer and incubated with FITC annexin V and propidium iodide (PI) solution for 15 min at room temperature in the dark. Cells were analysed by flow cytometry, and the results were expressed as the percentage of apoptotic cells, including both early (annexin V-positive, PI negative) and late (annexin V-positive, PI positive) apoptotic cells, relative to the total number of cells.

### Taqman low density arrays (TLDA) and relative quantification analysis

The expression of 43 genes selected in a previous study and involved in different biological processes in breast carcinogenesis was quantified by real time quantitative PCR using custom-made TaqMan low density arrays (TLDA, Applied Biosystems, Foster City, CA, USA)^27^. A qualitative review of the literature on breast cancer was conducted to select 43 genes involved in different biological processes in breast carcinogenesis (apoptosis, cell cycle, signalling pathways, angiogenesis, DNA repair, drug resistance) (Table 2). Two housekeeping genes were used as internal controls: *GAPDH* and *18S*. Gene expression analysis was performed as described previously^27^. Cells were cultured in 10 mm dishes and treated with 5 µM gefitinib and/or 100 nM everolimus for 48 h. Total RNA was extracted from cell lines using RNeasy Mini Kit according to the manufacturer’s instructions (Qiagen, Crawley, UK). The integrity and concentration of the RNA samples were assessed using an Agilent 2100 Bioanalyzer (Agilent Technologies, Foster City, CA, USA). Reverse transcription was performed with 1 µg of total RNA in a 20 µl reaction volume using the High Capacity cDNA Kit with RNase inhibitor according to the manufacturer’s instructions (Applied Biosystems, Foster City, CA, USA). Reaction conditions were 25 °C for 10 min, 37 °C for 120 min and 85 °C for 5 min. The resulting cDNA samples were mixed with 2X TaqMan Universal PCR Master Mix (Applied Biosystems). A total of 100 μl of each reaction mixture was loaded on the TLDA cards and centrifuged twice for 1 min at 1200 rpm. Cards were sealed, and the quantitative real-time PCR amplification was performed using an ABI Prism 7900 HT Sequence Detection System according to the manufacturer’s instructions (Applied Biosystems). Threshold cycle values (Ct) were determined with RQ Manager 1.2 software (Applied Biosystems). Each Ct value was normalized to the average Ct of *GAPDH* and *18S*. The relative quantification (RQ) of gene expression was determined using the comparative ΔΔCt method^29^. Unsupervised hierarchical clustering analysis based on ΔCt values was performed to identify differential gene expression profiles. Gene expression profiles were clustered using Euclidean distance and Ward’s method with SEM (Statistics Epidemiology Medicine) statistical software^30^.

**Table 2:** List of genes analyzed by TaqMan Low Density Array (TLDA).

Assay ID	Gene symbol	Gene name
Hs99999901_s1	*18S*	—
Hs99999905_m1	*GAPDH*	glyceraldehyde-3-phosphate dehydrogenase
Hs99999147_m1	*TP53*	tumor protein 53
Hs01076078_m1	*EGFR*	epidermal growth factor receptor
Hs00609566_m1	*IGFR*	insulin-like growth factor 1 receptor
Hs00179845_m1	*MET*	met proto-oncogene
Hs00176538_m1	*HER3*	human epidermal growth factor receptor 3
Hs00180679_m1	*PIK3CA*	phosphatidylinositol-4.5-bisphosphate 3-kinase
Hs00829813_s1	*PTEN*	phosphatase and tensin homolog
Hs00178289_m1	*AKT1*	v-akt murine thymoma viral oncogene homolog 1
Hs00234508_m1	*MTOR*	mechanistic target of rapamycin (serine/threonine kinase)
Hs00269944_m1	*BRAF*	v-raf murine sarcoma viral oncogene homolog B
Hs00364282_m1	*KRAS*	Kirsten rat sarcoma viral oncogene homolog
Hs01046830_m1	*MAPK1*	mitogen-activated protein kinase 1
Hs00385075_m1	*MAPK3*	mitogen-activated protein kinase 3
Hs00170630_m1	*FOS*	FBJ murine osteosarcoma viral oncogene homolog
Hs00153408_m1	*MYC*	v-myc avian myelocytomatosis viral oncogene homolog
Hs00153451_m1	*E2F1*	E2F transcription factor 1
Hs01548894_m1	*CDK2*	cyclin-dependent kinase 2
Hs00175935_m1	*CDK4*	cyclin-dependent kinase 4
Hs01026372_m1	*CDK6*	cyclin-dependent kinase 6
Hs00355782_m1	*CDKN1A*	cyclin-dependent kinase inhibitor 1A
Hs00923893_m1	*CDKN2A*	cyclin-dependent kinase inhibitor 2A
Hs00153277_m1	*CDKN1B*	cyclin-dependent kinase inhibitor 1B
Hs00175938_m1	*CDKN1C*	cyclin-dependent kinase inhibitor 1C
Hs00171105_m1	*CCNA1*	cyclin A1
Hs99999188_m1	*CCNB1*	cyclin B1
Hs00277039_m1	*CCND1*	cyclin D1
Hs00233356_m1	*CCNE1*	cyclin E1
Hs00967506_m1	*CHEK1*	checkpoint kinase 1
Hs00200485_m1	*CHEK2*	checkpoint kinase 2
Hs01078066_m1	*RB1*	retinoblastoma 1
Hs99999147_m1	*TP53*	tumor protein p53
Hs00180269_m1	*BAX*	BCL2-associated X protein
Hs00608023_m1	*BCL2*	B-cell CLL/lymphoma 2
Hs01018151_m1	*CASP8*	caspase 8
Hs00154260_m1	*CASP9*	caspase 9
Hs00234387_m1	*CASP3*	caspase 3
Hs00745222_s1	*XIAP*	X-linked inhibitor of apoptosis
Hs01075529_m1	*NOS2*	nitric oxide synthase 2. inducible
Hs01023894_m1	*CDH1*	cadherin 1, type 1, E-cadherin (epithelial)
Hs01547054_m1	*PLAU*	plasminogen activator. urokinase
Hs00900055_m1	*VEGF*	vascular endothelial growth factor A
Hs00234579_m1	*MMP9*	matrix metallopeptidase 9
Hs00173237_m1	*BRCA1*	breast cancer 1
Hs00242302_m1	*PARP1*	poly (ADP-ribose) polymerase 1
Hs00193931_m1	*PARP2*	poly (ADP-ribose) polymerase 2
Hs00184491_m1	*ABCB1*	ATP-binding cassette, sub-family B (MDR/TAP), member 1
Hs00184979_m1	*ABCG2*	ATP-binding cassette, sub-family G (BCRP1), member 2

### Statistical analysis

All experiments were repeated at least 3 times, except the gene expression experiments and western blots analyses, which were performed twice. All data are expressed as the mean ± SD. The statistical significance of differences between treated cells and untreated cells was assessed using two-way analysis of variance (ANOVA) followed by an unpaired Student’s t-test. A probability value p < 0.05 was considered significant.

Concerning the gene expression results, significant differences between the mean values of relative quantification (RQ) were evaluated only for genes that were 2-fold up- or downregulated after combination treatments. These differences were determined using an unpaired Student’s t-test between combination and everolimus (p/Evero) or gefitinib (p/Gef) treatments. A probability value p < 0.05 was considered significant.

## Results

### Growth inhibitory effect of everolimus and gefitinib in TNBC cell lines

**Figure 1:**
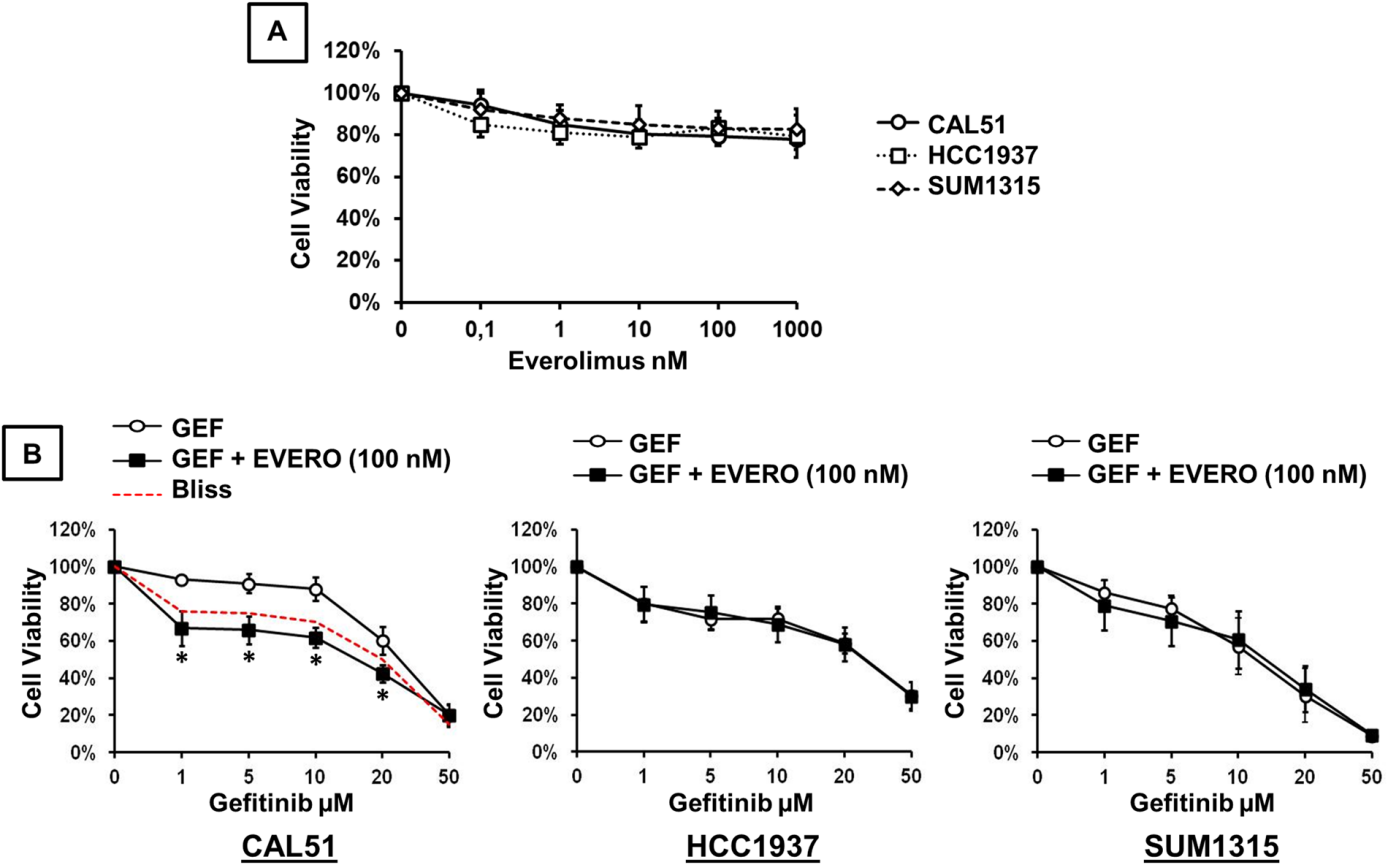
Cytotoxic effect of gefitinib and everolimus on TNBC cell lines. Cell viability assay was performed using the XTT assay as described in the methods section. (**A**) Cells were treated for 72 h with increasing concentrations of everolimus. (**B**) Cells were treated for 72 h with increasing concentrations of gefitinib (GEF) combined with 100 nM everolimus (EVERO). Results are expressed as percent of viability of untreated cell and are mean value ± SD of three independent experiments. The dashed red curve represents the expected Bliss values if the combined effects are additive. *p < 0.0001 for comparison between cells treated with gefitinib as a single agent (1, 5, 10, 20 and 50 μM) and cells treated with gefitinib (1, 5, 10, 20 and 50 μM) combined with 100 nM everolimus.

The growth inhibitory effects of everolimus and gefitinib were analysed by XTT assay in three TNBC cell lines. The drug concentrations used in this study are close to the median peak plasma concentrations reported in clinical pharmacokinetics studies^31–34^. We examined the *in vitro* sensitivity of TNBC cell lines to increasing concentrations (0.1, 1, 10, 100 and 1000 nM) of everolimus alone (Fig. 1A). When we exposed cells to everolimus at concentrations ranging from 0.1 to 1000 nM, cell viability was reduced by approximately 20% at the concentration of 100 nM. This growth inhibitory effect remained stable at higher concentrations. The concentration of everolimus required to reach the IC50 was higher than 1000 nM in the 3 TNBC cell lines. We then examined the *in vitro* sensitivity of TNBC cell lines to increasing concentrations of gefitinib combined with 100 nM everolimus. As shown in Fig. 1B, cell viability was reduced in a dose-dependent manner in all cell lines. When gefitinib was combined with 100 nM everolimus, no significant inhibition of cell proliferation was observed in HCC-1937 and SUM-1315 cells compared to that with gefitinib alone. Everolimus did not improve the effect of gefitinib in these two cell lines. By contrast, addition of everolimus in CAL-51 cells significantly increased the cytotoxic effect of gefitinib at concentrations ranging from 1 to 20 µM (p < 0.0001). Comparing the experimental and the Bliss theoretical curves, we observed a synergistic effect of combination treatments. The IC50 value of gefitinib alone in CAL-51 cells was 25.15 µM whereas the IC50 value of the combination with everolimus was 15.49 µM.

### Effect of everolimus and gefitinib on cell cycle distribution and apoptosis in TNBC cell lines

**Figure 2:**
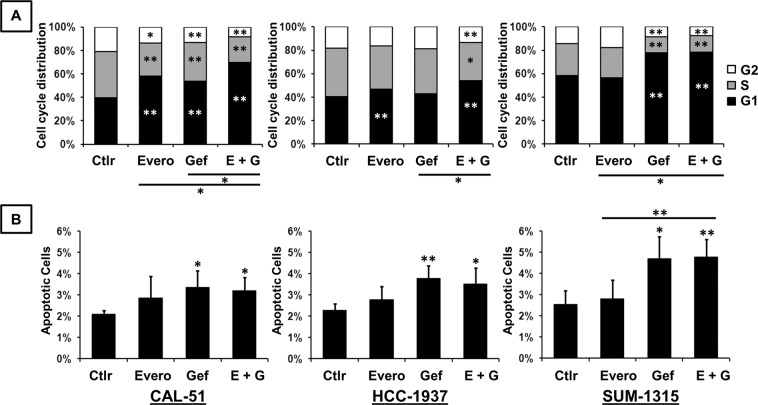
Effect of gefitinib and everolimus on cell cycle progression and apoptosis in TNBC cell lines. Cells were treated for 48 h with 5 µM gefitinib (Gef) and 100 nM everolimus (Evero), as single agents and in combination (E + G). Cells were stained with propidium iodide for analysis of cell cycle (**A**) and annexin V-FITC for analysis of apoptosis (**B**). Cell cycle distribution and quantification of positive cells were evaluated by flow cytometry. Data represent the mean value ± SD of triplicate experiments. *p < 0.05; **p < 0.01 for comparison between treated cells and untreated cells (Ctlr) using Student’s t-test.

We next investigated the effects of therapies on cell cycle distribution and apoptosis. Cells were treated for 48 h with 5 µM gefitinib and 100 nM everolimus as single agents and in combination. Analysis of cell cycle progression and apoptosis was performed by both annexin V-FITC and propidium iodide staining. Quantification of positive cells was evaluated by flow cytometry and expressed as a percentage of the total number of cells. In the CAL-51 cell line, everolimus and gefitinib, alone or in combination, significantly affected cell cycle distribution. We observed cell cycle arrest in the G1 phase, associated with a significant decrease in the proportion of cells in S and G2 phases, in treated cells compared with untreated cells (Fig. 2A). The combined treatment also induced a significant increase in the proportion of cells in the G1 phase compared with that with either everolimus or gefitinib given alone. Everolimus and gefitinib also induced cell cycle arrest in the G1 phase in HCC-1937 and SUM-1315 cells. Combination treatments also induced significant cell cycle arrest compared with that with either drug alone. Concerning the effect on apoptosis, only gefitinib and combined treatment induced significant cell death compared to that in the untreated control condition (Fig. 2B). Everolimus alone had no significant impact on apoptosis in all cell lines. In SUM-1315 cells, a significant increase in apoptotic cells was observed with combination treatments compared with that with everolimus alone. The combination treatments did not significantly increase apoptosis compared to that with gefitinib alone.

### Effect of everolimus combined with gefitinib on activation status of the key components of the RAS/MAPK and PI3K/AKT/mTOR signalling pathways

**Figure 3:**
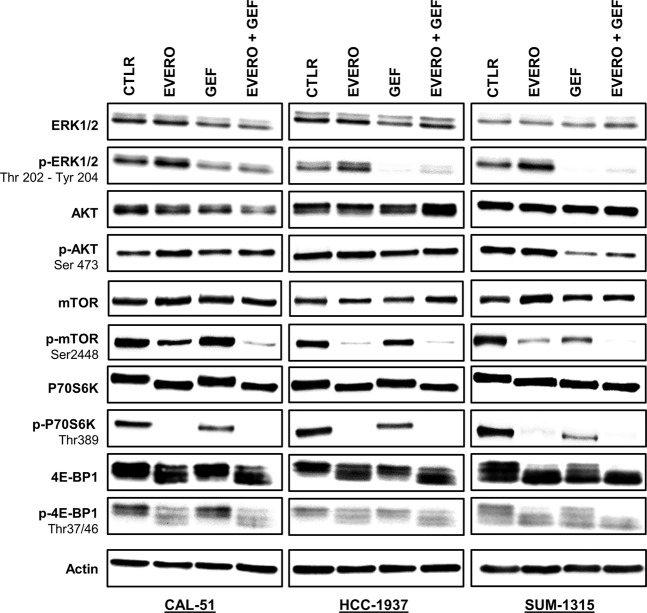
Western blot analysis of EGFR-dependent signalling pathways in TNBC cell lines treated with gefitinib and everolimus. Western blot analysis with the indicated antibodies was performed as described in the materials and methods section using 20 µg of whole cell protein extract. Cell lines were exposed for 24 h to 5 µM gefitinib (GEF) and/or 100 nM everolimus (EVERO). Beta-actin antibody was used as a loading control. Cropped blots from different gels are grouped together for clear illustration. The full-length gels are shown in the Supplementary Information. Data shown are representative of two independent experiments.

To assess the effects of gefitinib and everolimus on the PI3K/AKT/mTOR and RAS/MAPK signalling pathways, we analysed the protein expressions of total and phosphorylated forms of ERK, AKT, mTOR, P70S6K and 4E-BP1 by Western blotting. CAL-51, HCC-1937 and SUM-1315 cell lines were treated for 24 h with 5 µM gefitinib and 100 nM everolimus as single agents and in combination. Everolimus had no impact on the activation of ERK1/2, whereas gefitinib alone and combined treatment decreased the phosphorylation levels of ERK1/2 in all cell lines (Fig. 3). This effect was less marked in the CAL-51 cell line (1.9-fold and 1.8-fold, respectively after gefitinib and combination treatment exposure). In the HCC-1937 and CAL-51 cell lines, no effects were observed on phosphorylated form of AKT with the treatments. In SUM-1315 cells, only gefitinib alone and combined with everolimus clearly inhibited AKT phosphorylation (13.1-fold, respectively after combination treatment exposure). As expected, phosphorylation of mTOR was markedly reduced only in the presence of everolimus in all cell lines (1.9-fold, 19.9-fold and 5.5-fold, respectively in CAL-51, HCC-1937 and SUM-1315). Combined treatments synergistically inhibited mTOR phosphorylation in CAL-51 by 5.9-fold in comparison with cells treated with everolimus alone. In SUM-1315 cells, combined treatments completely abolished mTOR phosphorylation. Similarly, the P70S6K protein was completely dephosphorylated by everolimus as a single agent and in combination with gefitinib in all cell lines. In SUM-1315 and HCC-1937 cells, gefitinib alone was also able to reduce the phosphorylation of mTOR and P70S6K but less effectively than everolimus. Regarding 4E-BP1, everolimus clearly reduced the levels of the phosphorylated form only in CAL-51 cell line (1.6-fold and 2.1-fold, respectively after everolimus and combination treatment exposure). In the other two cell lines, everolimus had no effect or a weak effect on 4E-BP1 phosphorylation. Despite some differences, total forms of all these proteins appeared unaffected by everolimus or gefitinib.

### Altered expression of cell cycle-related genes and proteins in CAL-51 cells after everolimus and gefitinib treatments

**Figure 4:**
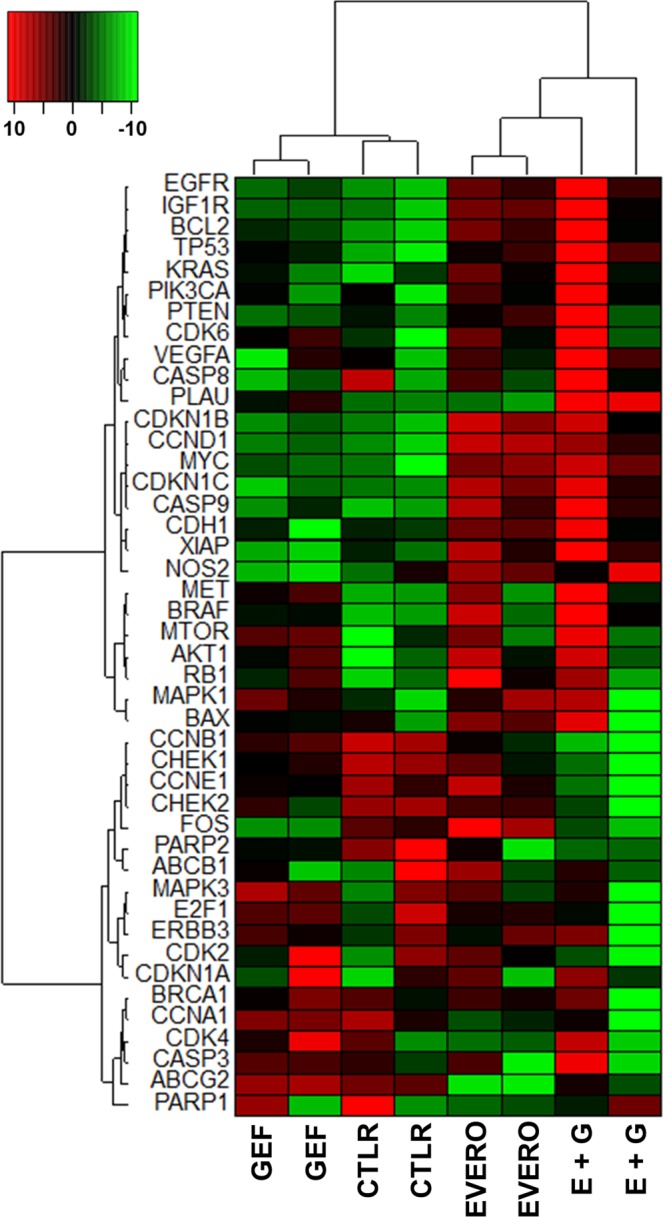
Unsupervised hierarchical clustering analysis showing the differentially expressed genes in CAL-51 cells treated with gefitinib and everolimus. The heatmap was generated by SEM statistical software according to ΔCt values (33). Genes in red and green indicate expression of upregulated and downregulated genes, respectively. Cells were treated or not (CTLR) for 48 h with 5 µM gefitinib (GEF) and 100 nM everolimus (EVERO), as single agents and in combination (E + G). Two independent experiments were performed.

Above, we showed a synergistic effect of combination treatments in cell proliferation and cell cycle experiments in the CAL-51 cell line. For further investigation, we analysed the expression of 44 genes involved in different biological processes (apoptosis, cell cycle, signalling pathways, angiogenesis, DNA repair, drug resistance) in this cell line after exposure to 5 µM gefitinib and 100 nM everolimus for 48 h. Hierarchical cluster analysis showed two distinct clusters allowing the differentiation of everolimus-treated cells from cells not treated with everolimus (Fig. 4). The data produced a dendrogram with cells falling into two groups characterized by different gene expression profiles. A cluster of 18 downregulated genes discriminated everolimus-treated cells from cells not treated with everolimus. This cluster included 8 genes involved in cell cycle control (*E2F1, CDK2, CDK4, CCNA1, CCNB1, CCNE1, CHEK1, CHEK2, CDKN1A*) and others genes related to DNA repair (*BRCA1, PARP1, PARP2*), drug resistance (*ABCB1, ABCG2*), and signalling pathways (*ERBB3, MAPK3, FOS*).

To assess the effect of the therapies on gene expression analysis, we calculated the relative quantification (RQ) value of each gene. Differentially expressed genes that were 2-fold up- or downregulated by a combination of everolimus plus gefitinib are summarized in Table 3. Results from the relative quantification (RQ) of gene expression analysis revealed significant differences in the expression of 6 cell cycle genes (Table 3). Combined treatment markedly decreased mRNA expression of checkpoint regulators *CHEK1* and *CHEK2*, as well as cyclin A1 (RQ = 0.4, p = 0.024), cyclin B1 (RQ = 0.3, p = 0.015) and cyclin E1 (RQ = 0.5, p = 0.050), thus inhibiting DNA damage repair and cell cycle progression. Another cell cycle-related gene, the cyclin-dependent kinase inhibitor gene *CDKN1C*, was significantly upregulated (RQ = 2.3, p = 0.048). Interestingly, among the genes of signalling pathways, four genes were upregulated by at least two-fold following combined treatment: *EGFR, IGFR, KRAS* and *BRAF*, but these differences did not reach statistical significance.

**Table 3:** Differentially expressed genes in CAL-51 cells treated with everolimus and gefitinib alone and in combination.

*Gene*^a^	Evero^b^	Gef^b^	E + G^b^	p /Evero^c^	p /Gef^c^
*EGFR*	1,0	0,5	2,0	*0,178*	*0,113*
*MYC*	1,2	0,5	1,7	*0,361*	*0,121*
*CDKN1C*	0,9	0,0	1,2	*0,474*	*0,048*
*CASP9*	0,7	0,4	1,1	*0,445*	*0,117*
*KRAS*	0,4	0,5	1,0	*0,503*	*0,556*
*IGFR*	0,5	0,3	1,0	*0,080*	*0,060*
*VEGFA*	0,1	0,2	1,0	*0,056*	*0,219*
*CDH1*	0,4	−0,3	1,0	*0,390*	*0,245*
*TP53*	0,2	0,5	0,9	*0,003*	*0,164*
*BRAF*	0,4	0,6	0,9	*0,493*	*0,435*
*CCNE1*	−0,3	−0,1	−1,0	*0,247*	*0,050*
*CHEK1*	−0,6	−0,3	−1,0	*0,357*	*0,086*
*CHEK2*	−0,6	−0,3	−1,0	*0,268*	*0,358*
*CCNA1*	−1,1	0,2	−1,2	*0,713*	*0,024*
*FOS*	0,8	−1,4	−1,4	*0,081*	*0,826*
*CCNB1*	−1,1	−0,4	−1,7	*0,148*	*0,015*

^a^List of genes that were 2-fold up- or downregulated after combination treatments.

^b^Data are presented as log2 of the average relative quantification after everolimus (100 nM) and gefitinib (5 µM) treatments as single agents (Evero; Gef) and in combination (E + G). A value of 1 corresponds to a two-fold upregulation and a value of −1 corresponds to a two-fold downregulation.

^c^Statistical differences between combination and everolimus (p/Evero) or gefitinib (p/Gef) treatments were assessed using Student’s t-test.

**Figure 5:**
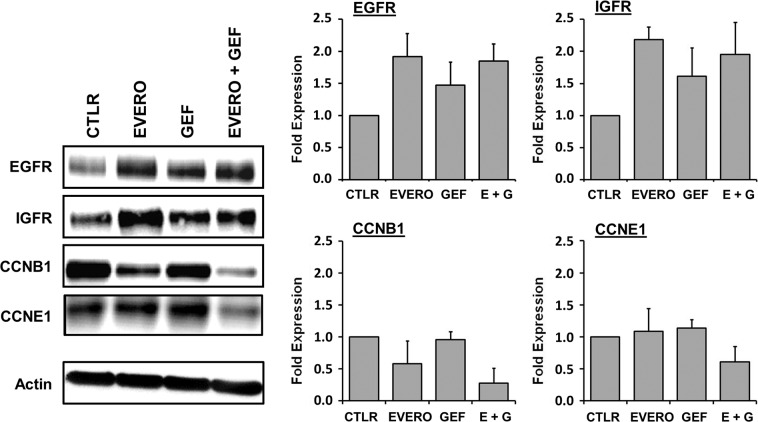
Western blot analysis of proteins encoded by genes differentially expressed in the CAL-51 cell line after exposure to gefitinib and everolimus. Cells were cultured for 48 h in the presence or absence (CTLR) of 5 µM gefitinib (GEF) and 100 nM everolimus (EVERO). Beta-actin antibody was used as a loading control. Cropped blots from different gels are grouped together for clear illustration. The full-length gels are shown in the Supplementary Information. Bar charts depict densitometric quantification of Western blot signals as described in the materials and methods section. Data shown are representative of two independent experiments.

The expression of several proteins encoded by these genes was also analysed in CAL-51 cells via Western blotting (Fig. 5). Similar to the gene expression analysis, the Western blot analysis showed that combined therapy reduced the expression levels of cyclin B1 and cyclin E1, compared to those with monotherapy. The combination treatment exposure reduced cyclin B1 expression by up to 2.5-fold and 5-fold, compared with that with everolimus and gefitinib as single agents, respectively. Cyclin E1 was downregulated by 2-fold after combination treatment compared with monotherapy. Western blotting results also confirmed increased expression of the tyrosine kinase receptors EGFR and IGFR after treatment. Everolimus and gefitinib, as single agents or in combination, increased the expression of EGFR and IGFR by up to 2-fold compared with that in the untreated control group.

## Discussion

Triple-negative breast cancer is more aggressive than other types of breast cancers and remains the most deadly of all breast cancer types. Currently, approximately 15–20% of breast cancers are triple-negative and the treatment of patients with TNBC still presents a major challenge in breast cancer research^35^. TNBC is generally characterized by EGFR overexpression and frequent dysregulation of the PI3K/AKT/mTOR signalling pathway. These features are considered potential therapeutic targets in TNBC^17^. Recently, we investigated the *in vitro* effect of various EGFR inhibitors in cultured TNBC cell lines. We reported that anti-EGFR agents alone inhibited the RAS/MAPK signalling pathway more effectively than the PI3K/AKT/mTOR pathway^26,27^. These data provide evidence that the efficacy of anti-EGFR targeted therapies in TNBC can be impaired by alterations in the PI3K/AKT/mTOR axis.

In the present study, we provided lines of evidence supporting the rationale for combining the mTOR inhibitor everolimus with gefitinib for TNBC with genetic alterations in the PI3K/AKT/mTOR signalling pathway. We examined the antitumour activity of these targeted therapies in TNBC cell lines that overexpress EGFR and do or do not harbour genetic alterations in PI3K/AKT/mTOR signalling pathway. The CAL-51 cell line harbours an activating mutation in PI3K (E542K), the gene that encodes the p100α catalytic subunit of PI3K, and homozygous deletion of *PTEN*, leading to the constitutive activation of the PI3K/AKT/mTOR signalling pathway^24^. The E542K mutation occurs in approximatively 8% of TNBC. The HCC-1937 cell line also harbours homozygous deletion of the *PTEN* gene. The SUM-1315 cell line has no mutational activation of the EGFR signalling pathways.

We demonstrated that everolimus enhances the growth inhibitory effect of gefitinib at all concentrations tested in CAL-51 cells. We also observed that the combination treatment induced cell cycle arrest at the G1/S checkpoint and significantly increased apoptosis in this cell line. The cell cycle perturbations observed using flow cytometry are in accordance with the results from the gene expression analysis. Hierarchical cluster analysis grouped cells in two different clusters according to the presence or absence of everolimus. These two groups were characterized by different gene expression profiles including genes involved in cell cycle control such as *CCNB1, CCNE1, CHEK1*, and *CHEK2*. In addition, protein expression analysis showed downregulated expression of cyclin E1 and cyclin B1 which are two key molecules for G1/S and G2/M transition during the cell cycle. Collectively, these results suggest that the potential benefit of combination therapy with everolimus and gefitinib might be restricted to mutant *PI3K* cells.

Several studies have reported that simultaneous inhibition of EGFR and mTOR could provide a synergistic antitumour effect in various human cancers, including TNBC^20–23^. *In vitro* studies have shown that everolimus enhances the efficacy of gefitinib in gefitinib-resistant NSCLC cells^21,36^. Evidence supports the dysregulation of EGFR downstream signalling pathways, including *PI3K* or *PTEN* mutations, as a possible mechanism of primary resistance^37,38^. Activating mutations of *PI3K* lead to persistent activation of the PI3K/AKT/mTOR pathway despite EGFR inhibition and have been shown to confer resistance in gefitinib-sensitive lung cancer cell lines^39^. An *in vitro* study investigated the combined inhibitory effect of everolimus and gefitinib in EGFR wild-type NSCLC cell lines with or without *PI3KCA* mutation. Consistent with our result, the authors demonstrated that concomitant inhibition of the EGFR and mTOR proteins induced cell proliferation inhibition and cell cycle arrest in EGFR-wild-type NSCLC cells with a *PI3KCA* mutation^20^. Other preclinical studies showed that simultaneous targeting of EGFR and mTOR with the tyrosine kinase inhibitor erlotinib and rapamycin, respectively, inhibited the activation of the PI3K/AKT/mTOR signalling pathway, resulting in the inhibition of cell proliferation and cell cycle progression in colorectal carcinoma cells^22,40^.

Moreover, Lehmann et *al*. previously reported that TNBC cell lines with activated PI3K/AKT signalling due to *PI3KCA* mutations or *PTEN* deficiency are highly sensitive to dual PI3K/mTOR inhibition^24^. The authors demonstrated that *PI3KCA* mutations predicted sensitivity to dual PI3K/mTOR inhibition, but *PTEN* deficiencies did not correlate with sensitivity. The PTEN protein blocks PI3K and negatively regulates the PI3K/AKT/mTOR pathway. Loss of PTEN expression leads to activation of this pathway by persistent signalling through AKT^41,42^. A further activating mutation of *PI3KCA* amplifies the activation of AKT and the resistance to EGFR inhibitors. The presence of coexisting mutations in *PI3KCA* and *PTEN* interferes with the efficacy of EGFR inhibitors. In our study, we showed that dual inhibition of mTOR and EGFR induced a synergistic antiproliferative effect in the *PI3KCA* and *PTEN*-mutant CAL-51 cell line, but not in the *PTEN*-null HCC-1937 cell line. The *PI3KCA* mutation could thus predict sensitivity to the mTOR inhibitor everolimus combined with gefitinib in TNBC.

Reports from other authors have shown that lapatinib, a dual inhibitor of EGFR and HER2, combined with the mTOR inhibitor rapamycin induced significant *in vitro* growth inhibition of TNBC cell lines and synergistically reduced tumour growth in TNBC cell-derived xenograft models^23^. According to the authors, the synergistic effects observed with combination therapy were due, at least in part, to a decrease in AKT activation induced by mTOR inhibitors. Indeed, it has been reported that mTOR inhibition can lead to activation of the PI3K/AKT/mTOR signalling pathway through activation of AKT, possibly induced by a feedback mechanism^43,44^. They also demonstrated that EGFR inhibition abrogated rapamycin-induced AKT activation in TNBC cells. Consistent with these results, we observed an increase in AKT phosphorylation in the CAL-51 cell line when cells were treated with everolimus alone. This activation of AKT was then reversed by the addition of gefitinib (Fig. 3). Furthermore, it has been reported that phospho-AKT is highly expressed in TNBC and is strongly correlated with loss of *PTEN* expression and/or activating mutations in *PI3K*^45^.

## Conclusions

In conclusion, the combination of gefitinib and everolimus may provide a potential therapeutic option for EGFR-expressing and PI3K-mutated TNBC patients. This therapeutic approach is interesting as activating mutations in *PI3KCA* are the most frequent next to *TP53* mutations in TNBC^13,46,47^. Although *in vivo* studies are needed to confirm these results, our findings provide preclinical evidence supporting the rationale for combining the mTOR inhibitor everolimus with gefitinib for the treatment of TNBC with genetic alterations of the PI3K/AKT/mTOR signalling pathway. This combination therapy warrants further investigation in patients with TNBC through a prospective clinical trial.

## Supplementary information

Supplementary Information.

## Data Availability

The datasets generated or analysed during the current study are available from corresponding author upon reasonable request.
