# Supplementary material for: Co-targeting EGFR and mTOR with gefitinib and everolimus in triple-negative breast cancer cells
Source: Sci Rep. 2020 Apr 14;10:6367. doi: 10.1038/s41598-020-63310-2 (PMC7156377; doi:10.1038/s41598-020-63310-2)
Supplement: Supplementary file 1 — Supplementary Information. [file 41598_2020_63310_MOESM1_ESM.pdf]

Supplementary Information

Co-targeting EGFR and mTOR with gefitinib and everolimus in triple-negative breast cancer cells

Abderrahim El Guerrab, Mahchid Bamdad, Yves-Jean Bignon, Frédérique Penault-Llorca and Corinne Aubel

Full-length Western blots are shown in the same order as they appear in the main manuscript.

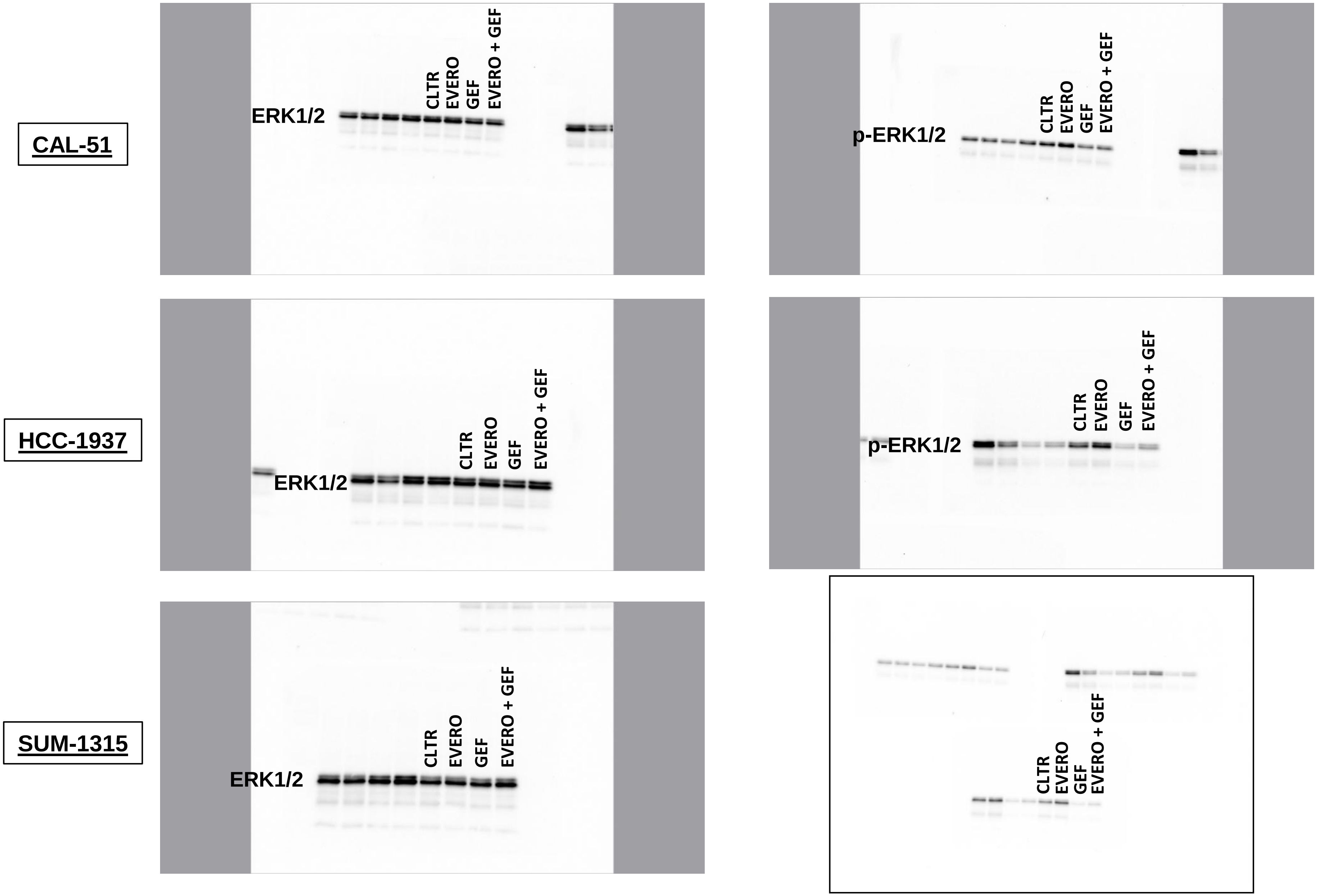

Full-length blots – ERK and phospho-ERK – Figure 3

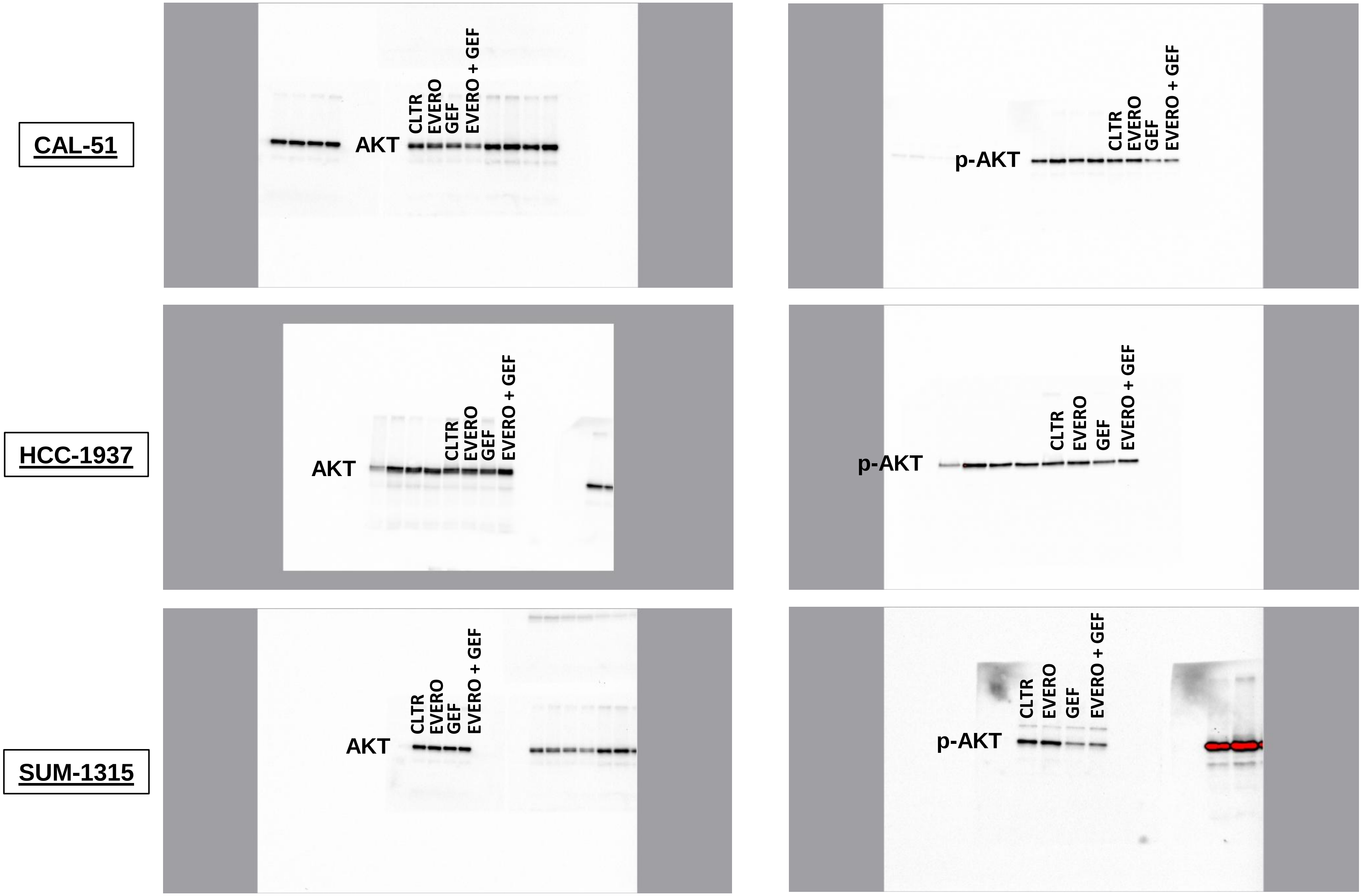

Full-length blots – AKT and phospho-AKT – Figure 3

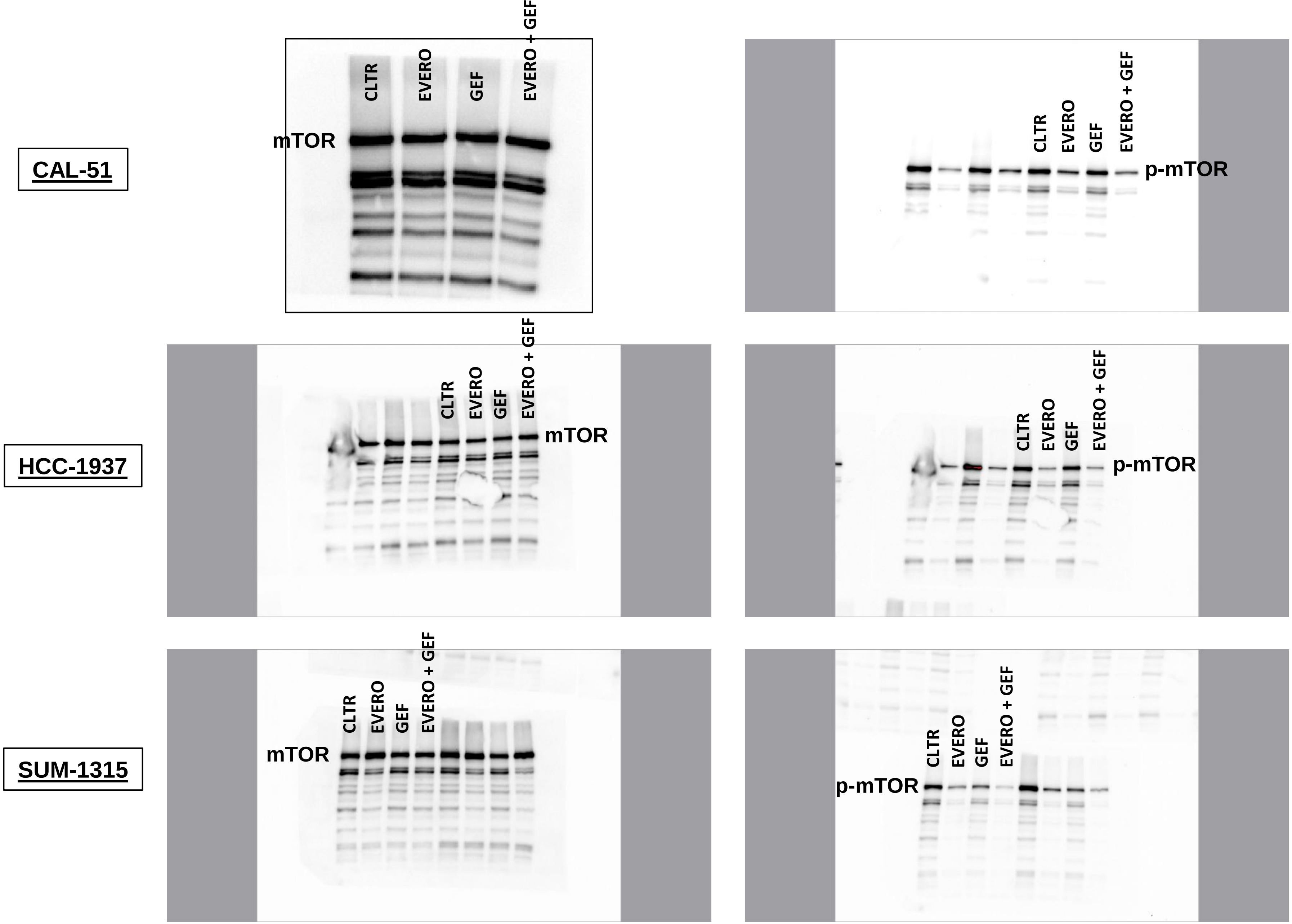

Full-length blots – mTOR and phospho-mTOR – Figure 3

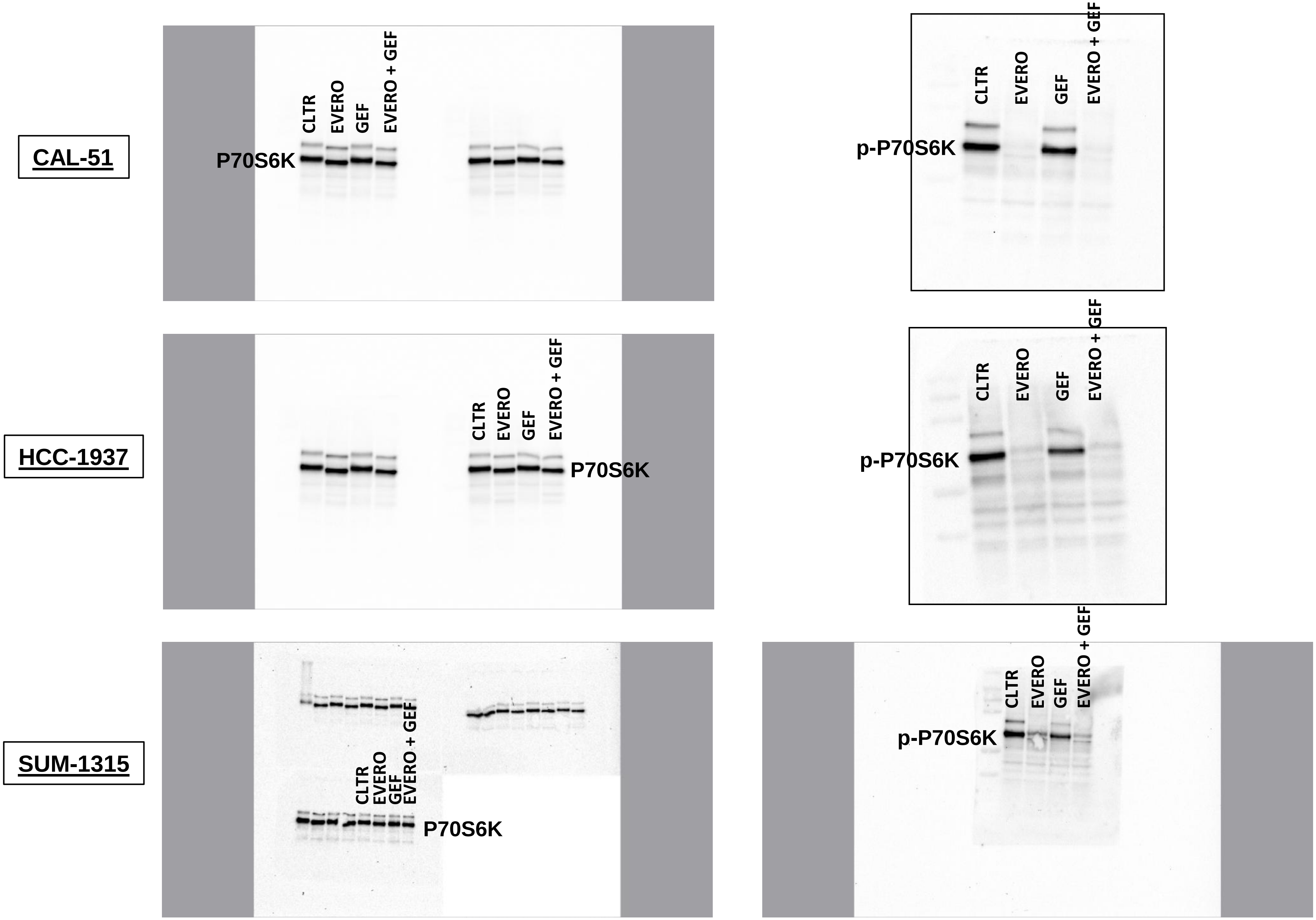

Full-length blots – P70S6K and phospho-P70S6K – Figure 3

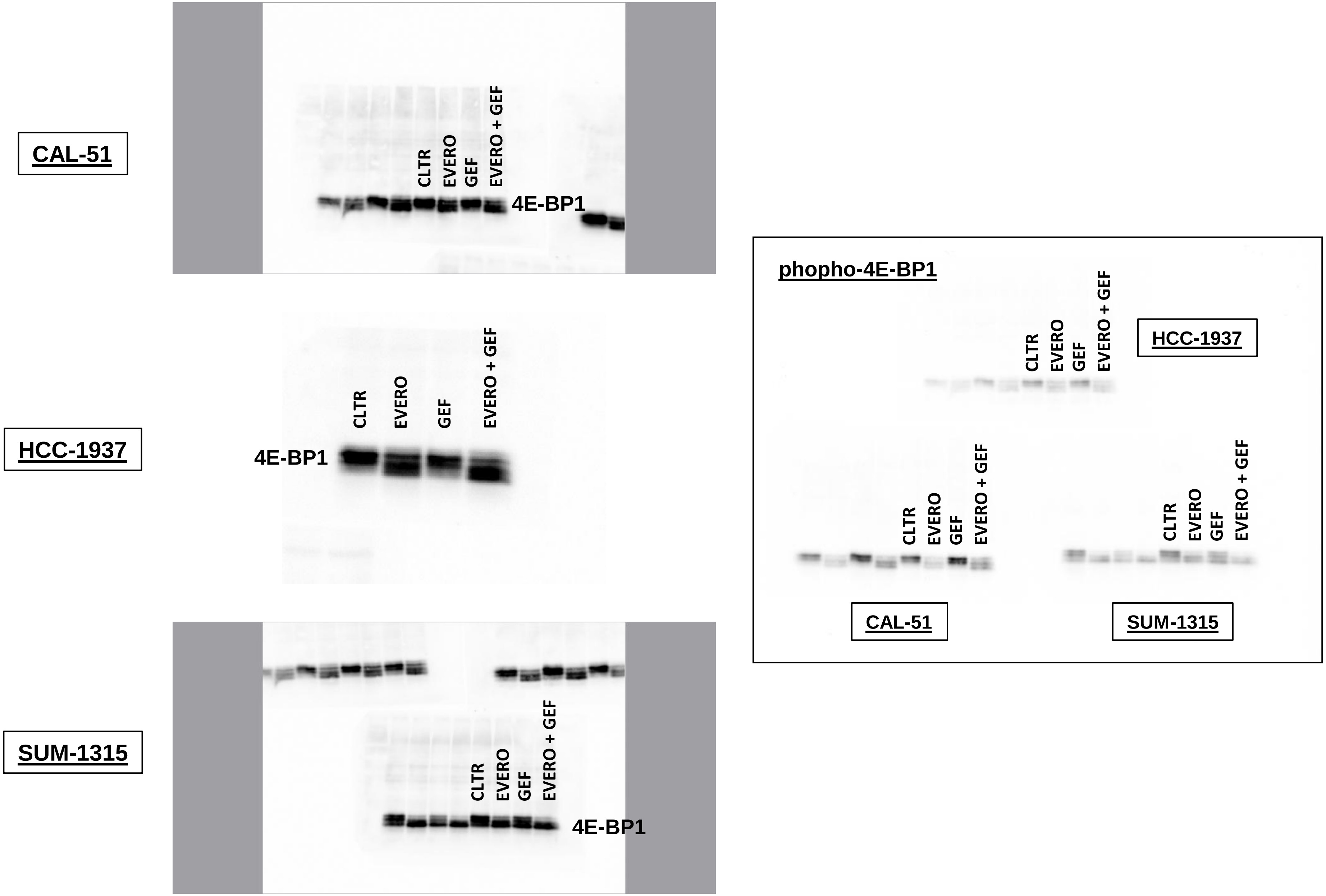

Full-length blots – 4E-BP1 and phospho-4E-BP1 – Figure 3

CAL-51

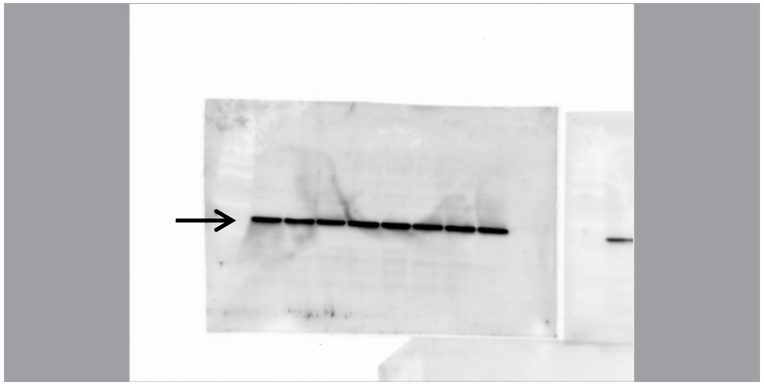

HCC-1937

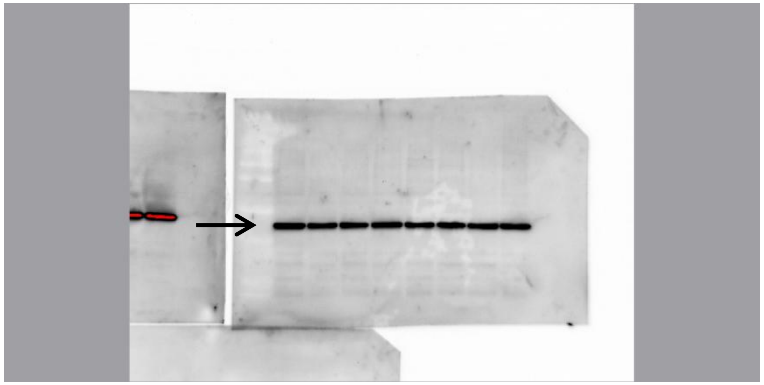

SUM-1315

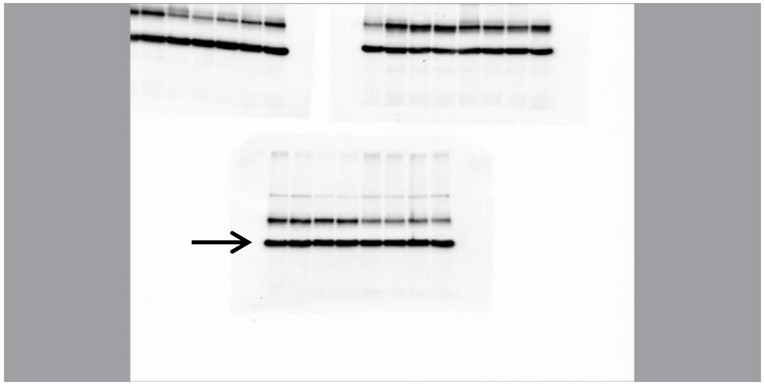

Full-length blots – Actin – Figure 3

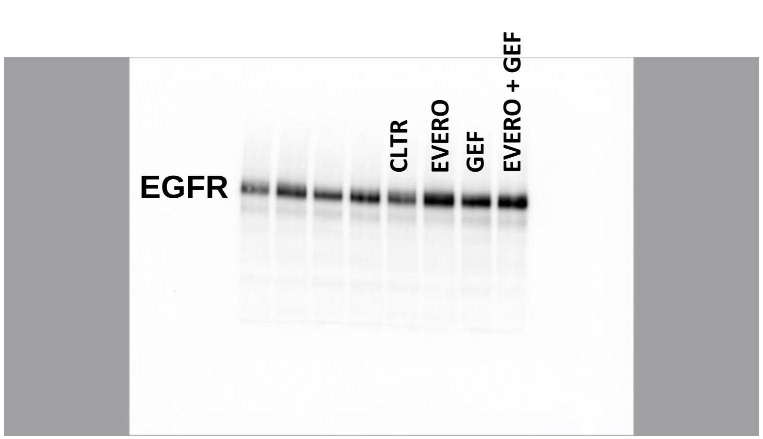

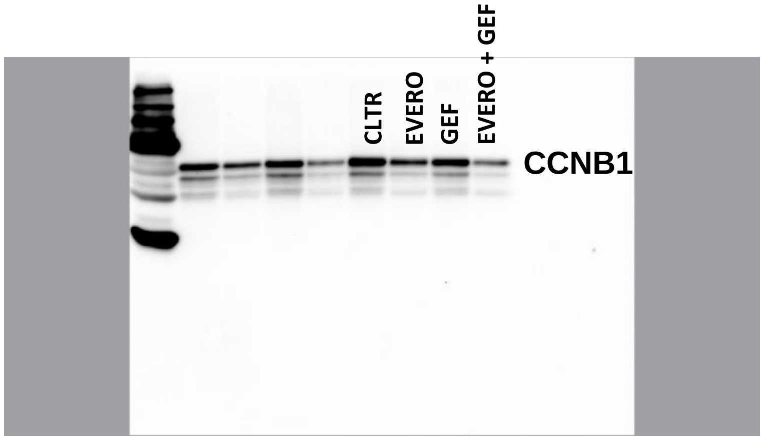

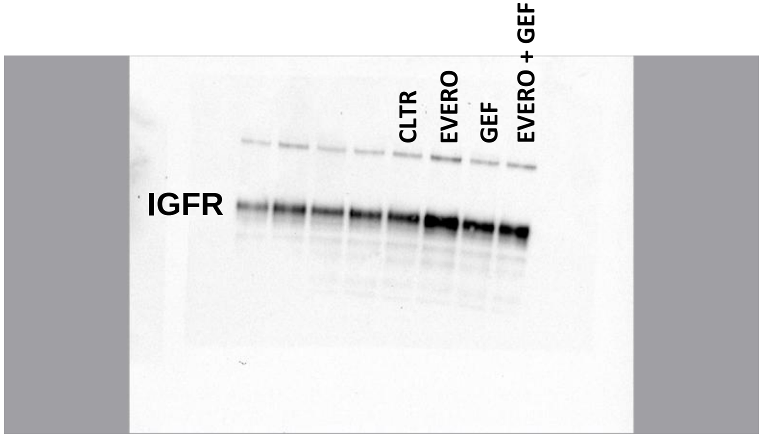

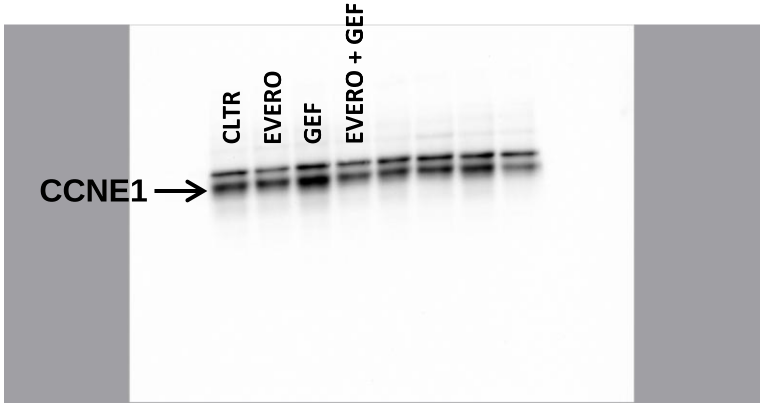

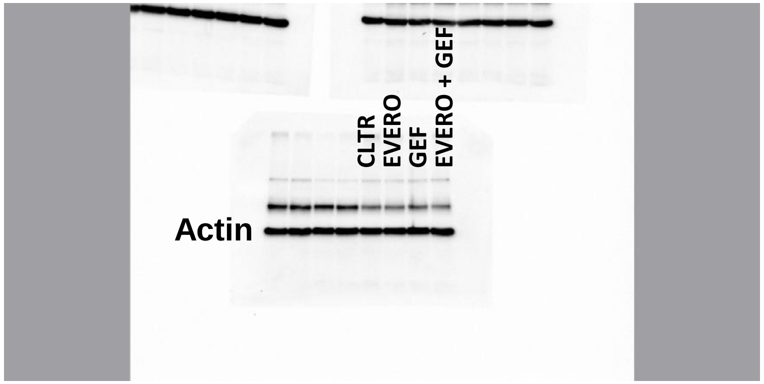

<u>**Full-length blots of Figure 5**</u>
